# Cerebral sparganosis in children: epidemiological, clinical and MR imaging characteristics

**DOI:** 10.1186/1471-2431-12-155

**Published:** 2012-09-24

**Authors:** Caigui Gong, Weihua Liao, Ashley Chineah, Xiaoyi Wang, Bob L Hou

**Affiliations:** 1 Department of Radiology, Xiangya hospital, Central South University, Changsha, Hunan, 410008, China; 2 Center of Advanced Imaging, Department of Radiology, West Virginia University, Morgantown, WV, 26506, USA

## Abstract

**Background:**

Cerebral sparganosis in children is an extremely rare disease of central nervous system, and caused by a tapeworm larva from the genus of Spirometra. In this study, we discussed and summarized epidemiological, clinical and MR imaging characteristics of eighteen children with cerebral sparganosis for a better diagnosis and treatment of the disease.

**Methods:**

Eighteen children with cerebral sparganosis verified by pathology, serological tests and MR presentations were retrospectively investigated, and the epidemiologic and clinical characteristics of the disease were studied.

**Results:**

Twenty-seven lesions were found in the eighteen children. Twelve lesions in twelve patients were solitary while the lesions in the rest six patients were multiple and asymmetrical. The positions of the lesions were: seven in frontal, eleven in parietal, four in temporal and two in occipital lobes, one in basal ganglia, one in cerebella hemisphere and one in pons. The lesions were presented as slight hypointensity on T1-weighted images but moderate hyperintensity on T2-weighted images with perilesional brain parenchyma edema. Enhanced MR scans by using Gadopentetic Acid Dimeglumine Salt were performed in the patients, and the images demonstrated abnormal enhancements with the patterns of a peripheral ring, or a tortuous beaded, or a serpiginous tubular shape. Follow-up MR scans were preformed for eight patients, and three out of the eight cases exposed migrations and changes in shapes of the lesion areas.

**Conclusions:**

The MR presentations in our study in general were similar to those in previous studies. However serpiginous tubular and comma-shaped enhancements of lesions have not been previously reported. The enhanced MR imaging and follow-up MR scans with the positive results from serological tests are the most important methods for the clinical diagnosis of cerebral sparganosis in children.

## Background

Cerebral sparganosis in children is a very rare parasitic disease of central nervous system, and caused by the infection of a migrating plerocercoid larva. Cases of cerebral sparganosis have been mostly reported in southeastern Asia [1-5]. In 1918, Takeuchi et al. reported the first such case [2]. In China, the disease is mainly in Shanghai city, and provinces of Guangdong, Taiwan, Sichuan and Fujian. The highest incidence was found in the ages from ten to thirty [6]. Since it is a rare disease, there were few published papers with always few patient cases related to MRI diagnosis of the disease [7-9]. Hence, to have a study including more cases can help us to have a comprehensive and profound understanding on the MRI characteristics of cerebral sparganosis in children.

For this purpose, we presented a retrospective study of eighteen children with cerebral sparganosis verified by pathology, serological tests and MR images. These data were collected from January 1993 through June 2008. In this paper, we discussed the causes and clinical presentation of the disease, explored clinical significance of MR protocols, and investigated MRI characteristics of the disease for a better diagnosis and treatment.

## Methods

### Patients

The patients were consented prior to the MRI scans. Ten males and eight females patients with the age of 9.00 ± 4.01 year old and the age range from three to seventeen years old were verified to have cerebral sparganosis by pathological, serological tests and MR images, and were included in this retrospective study.

Examinations of cerebrospinal fluid cytology in four cases revealed a mild elevation of cell count with raising lymphocytic or eosinophilic count and activated lymphocytes and monocytes. Enzyme-linked immunosorbent assay (ELISA) of cerebrospinal fluid or blood was detected [10], and the results demonstrated anti-sparganosis and antibody for all eighteen cases. Among the cases, six (33.33%) had experienced high risk factors, such as a history of eating uncooked frog meat or ingesting uncooked crabs or drinking contaminated water. Four cases (22.22%) were misdiagnosed as brain tumors and underwent surgeries. A white worm was found in each of two patients’ brains during the surgeries.The rest fourteen patients were treated with praziquantel for deworming. For the 18 patients, they were diagnosed by ELISA of cerebrospinal fluid or blood, cerebral MRI, history of risky behavior, the treatments of deworming or surgery. Considering that the chemiluminescence ELISA demonstrats high sensitivity and specificity for human sparganosis mansoni, we think the combination information with MRI and the positive ELISA results is a reliable diagnosis for cerebral sparganosis.

Health conditions in twelve patients after the treatments including all four resections were improved. No obvious changes in health conditions in five patients were noted, but deworming treatment was continued after discharge of the patients. A slight deterioration in clinical condition in a patient was noted.

In Table 1, we listed the ingestion of pathways, clinical symptoms, treatment, ELISA data and MRI information of the eighteen patients. Consent was also obtained from the parents or guardians of the children for each presenting clinical examination.

**Table 1:** Clinical, treatment, ELISA and MRI information for 18 children with cerebral sparganosis

**Case**	**Age/sex**	**Ingestion of pathways**	**Presenting symptom(s)**	**Results of ELISA**		**Treatment**	**MRI follow-up period (m)**	**MRI enhancement Patterns**
				**Serum**	**CSF**			
1	6/F	Frog (2y ago)	Headache, altered mental status	+	+	Praziquantel	NF	tortuous linear shaped enhancement
2	3/M	NI	Seizures	+	+	Degenerated worm removed	NF	serpiginous tubular enhancement
3	5/M	NI	Hemiparesis, dizziness	+	+	Praziquantel	2	beaded enhancement
4	11/M	NI	Headache, seizures	+	+	Praziquantel	NF	ring enhancement
5	14/F	Frog (6y ago)	Altered mental status	+	+	Praziquantel	6	tortuous linear shaped enhanceme
6	4/F	Untreated water (for 1y)	Hemiparesis	+	+	Removal of live worm	NF	ring enhancement
7	7/M	NI	Headache, blurred vision, palsy	+	+	Praziquantel	1	beaded enhancement
8	13/M	uncooked crabs (8y ago)	Seizures, altered mental status	+	+	Praziquantel	5	beaded enhancement
9	15/M	NI	Headache, projectile vomiting	+	+	Degenerated worm removed	NF	ring enhancement
10	9/F	NI	Hemiparesis	+	+	Praziquantel	7	comma shaped and ring enhancement
11	12/M	uncooked crabs (6y ago)	Seizures, hemiparesis	+	+	Praziquantel	NF	beaded enhancement
12	9/F	NI	Headache, intermittent seizures	+	+	Live worm removed	7	serpiginous tubular enhancement
13	9/M	NI	Headache, seizures, blurred vision	+	+	Praziquantel	5	nodular enhancement
14	10/M	NI	Headache, altered mental status	+	+	Praziquantel	NF	beaded enhancement
15	7/F	NI	Seizures	+	+	Praziquantel	3	beaded enhancement
16	5/M	NI	Seizures, dizziness	+	+	Praziquantel	NF	serpiginous tubular enhancement
17	6/F	NI	Altered mental status	+	+	Praziquantel	7	nodular enhancement
18	17/F	Untreated water (for 5y)	Seizures	+	+	Praziquantel	NF	beaded enhancement

**Note:***NI* = no ingestion; + = positive; - = negative; *NF* = no follow-up.

### Magnetic resonance scans

The MR scans were performed by using two Siemens scanners. Sixteen patients were scanned in a 1.5 T scanner with a quadrature head coil by applying following pulse sequences and acquisition parameters. A spin-echo T1-weighted (axial, TR = 450 ms and TE = 10 ms), a turbo spin-echo T2-weighted (axial, TR = 4200 ms and TE = 98 ms), and a fluid attenuated inversion recovery (axial, TR = 8500 ms, TE = 107 ms and TI = 110 ms) scans were preformed for pre-contrast images. After an intravenous injection (0.1 mmol/kg) of Gadopentetic Acid Dimeglumine Salt (Magnevist; Bayer Schering Pharma AG, Berlin, Germany) an axial, sagittal and coronal spin echo T1-weighted images (TR = 450 ms and TE = 10 ms) were obtained. For all scans, the slice thickness was 5 mm with a gap of 1.5 mm, the acquisition matrix was 210 × 240, and the number of slices was 19. We also performed MR examinations with a 1.0 T scanner with a quadrature head coil for the rest two patients by using the following sequences and parameters. A spin-echo T1-weighted (axial, TR = 600 ms and TE = 15 ms) and a turbo spin-echo T2-weighted (axial, TR = 2300 ms and TE = 90 ms) sequences were applied for pre-contrast MR images. After an intravenous injection (0.1 mmol/kg) of Gadopentetic Acid Dimeglumine Salt (Magnevist; Bayer Schering Pharma AG, Berlin, Germany) an axial, sagittal and coronal spin-echo T1-weighted images (TR = 600 ms and TE = 15 ms) were obtained. For all scans, the slice thickness was 5 mm with a gap of 1.5 mm, the acquisition matrix was 230 × 230, and the number of slices was 19. Retrospective diagnoses of MR images of the eighteen cases of cerebral sparganosis were carried out by two coauthors (neuroradiologists with more than five years experience) by filling a specifically designed form independently. The information in the forms includes locations, shapes, numbers, enhancement shapes and regions of the lesions. If the findings were different between the neuroradiologies, a consensus was sought. There were pathology reports for four cases after surgeries, and eight patients were scanned again in follow-up MRI.

## Results

### MR presentations

Among the eighteen patients, we found totally twenty-seventh lesions. Twelve patients had solitary (66.67%) lesion, and six had multiple lesions (33.33%). Six patients had different degrees of mass effect. The lesions positions include eleven (40.74%) in parietal lobes (examples shown in Figures of 1, 2, 3, 4 and 5), seven (25.92%) in frontal lobes (an example shown in Figure 6), four (14.81%) in temporal lobes, two (7.41%) in occipital lobes, one (3.70%) in basal ganglia, one (3.70%) in cerebella hemisphere and one (3.70%) in pons. Fourteen (51.85%) lesions were located in white matter, nine (33.33%) in junctions of gray-white matter and four (14.81%) in gray matter of cerebrum. The lesions were slight hypointensity on T1 weighted images (T1WI), but moderate hyperintensity on T2 weighted images (T2WI). The shapes of the lesions were irregular patchy and or serpiginous tubular (an example shown as the black arrows in Figures of 3A and 3B) and or tortuous linear. In the perilesional parenchyma, an edema with irregular large patchy could be appreciated (the white arrow in Figure 3A).

**Figure 1:**
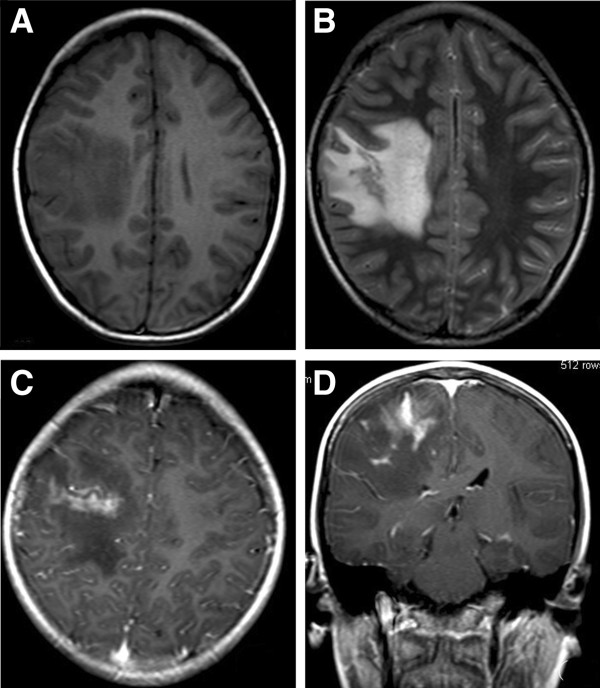
**MR images for the case 2. A**. The T1W image shows there is an irregular large patchy area of hypointensity in the right parietal lobe. **B**. The T2W image shows there is a serpiginous tubular hyperintense lesion (Black arrow) with a perilesional large patchy irregular edema (White arrow) in the right parietal lobe. The edema shows low signal on the T1W image and high signal on the T2W image. **C** and **D**. The enhanced T1W images show a serpiginous tubular enhancement for the lesion.

**Figure 2:**
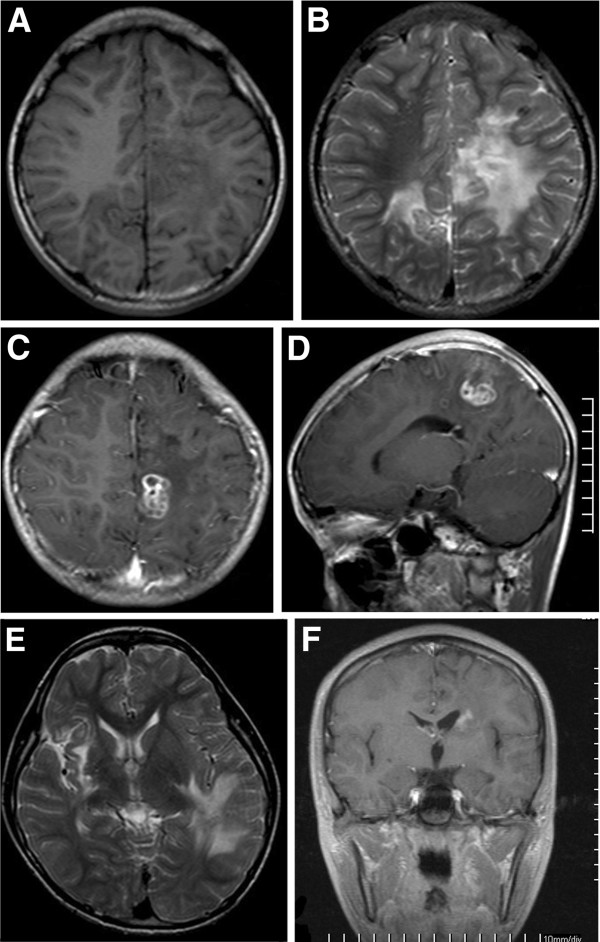
**MR images for the case 8. A**. The T1W image shows there are two white matter irregular patchy with hypointensity in the parietal lobes. **B**. The T2W images shows there are two white matter irregular patchy with hyperintensity in the parietal lobes. **C** and **D**. The enhanced T1W images show a beaded enhancement for the lesion. **E** and **F**. The images from the MR follow-up scans after 5 months shows the location and shape for the enhancing lesion was changed (**C** and **E**).

**Figure 3:**
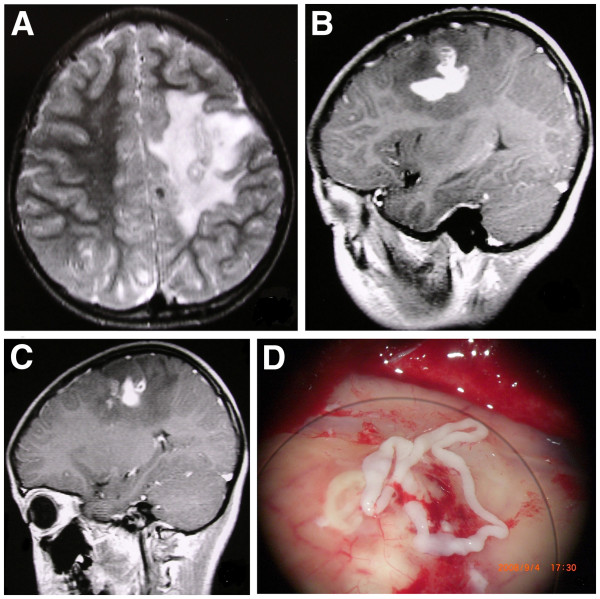
**MR images for the case 12. A**. The T2W image shows there is a serpiginous shape with inhomogeneous slight hyperintensity (Black arrow) in the left parietal lobe including mainly white matter area. Irregular large patchy edema can be seen in the perilesional parenchyma (White arrow) which is high signal on the T2W image. **B**. The enhanced T1W image shows a serpiginous tubular enhanced lesion with a diameter of 6.1 cm (which is the longest in the study). **C**. The enhanced T1W image from the MR follow-up after 7 months shows change in location and shape for the enhanced lesion. The lesion area was slightly moved medially and posteriorly, and appears as a comma shaped and ring enhancement. **D**. After the surgery, the worm was still alive, and its length was 13 cm.

**Figure 4:**
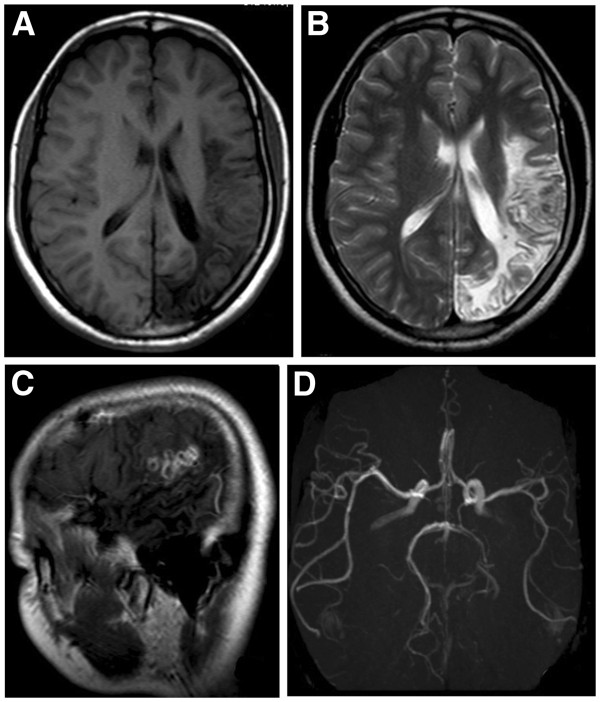
**MRI images for the case 15 in the three-month follow-up. A**. The T1W image shows there is an irregular large patchy area with hypointensity in the left parietal lobe including white matter, gray-white matter junction, and gray mater areas. Parenchymal atrophy was seen as decrease in the size of ipsilateral gyrus and sulcus, but increase in the size of corresponding fissures of the lesion and the left ventricle. **B**. The T2W image shows there is an irregular large patchy area with hyperintensity in the left parietal lobe including white matter, gray-white matter junction, and gray mater areas. Atrophy of parenchyma is seen as the same as seen in the T1W image. **C**. The enhanced T1W image shows a few beaded enhancing lesions. **D**. The MRA slice shows decrease in the size and number of the branches in the left posterior artery.

**Figure 5:**
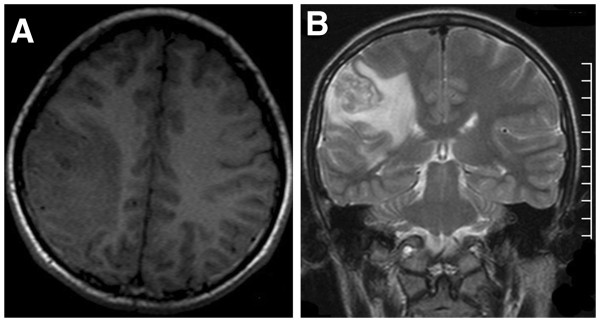
**MRI images for the case 9. A**. The T1W image shows there is an irregular large patchy area with hypointensity in the right parietal lobe including white matter, gray-white matter junction, and gray mater areas. **B**. The T2W image shows there is an irregular large patchy area with hypointensity (Black arrow) in the right parietal lobe including white matter, gray-white matter junction, and gray mater areas. Large patchy areas of the edema was seen in the area surrounding the lesion (White arrow). The edema on a T1W image appears as low signal but on a T2W image is high signal. **C**. The enhanced T1W image shows a ring-enhanced lesion. **D**. is the microscopic appearance of post surgery of the cerebral sparganosis (HE × 200). Pathological diagnosis proved cavitations due to fibroplasias of cerebral tissue with degenerated larva, partly necrotized substances and calcification. There was fibrosis of surrounding the brain tissue with several granulomas and infiltration of inflammatory cells and gliosis. These findings highly suggested granulomas due to parasitic infection.

**Figure 6:**
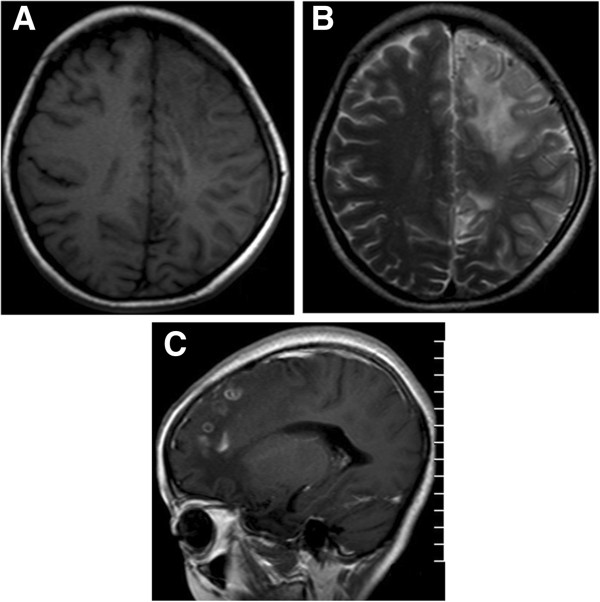
**MR images for the case 4. A**. The T1W image shows there is an irregular large patchy area with hypointensit in the left frontal lobe including the areas of the white matter, gray-white matter junction, and gray mater. **B**. The T2W image shows there is an irregular large patchy area with hyperintensity in the left frontal lobe including the areas of the white matter, gray-white matter junction, and gray mater. **C**. The enhanced T1W image shows a few ring-enhanced lesion areas.

The enhanced scans by using the contrast agent were performed in these 18 patients, and all lesions showed different enhanced shapes. Table 1 listed the MRI enhancement patterns of the eighteen patients. Three cases (16.67%) showed a ring (examples shown in Figures of 6C and 6C), seven (38.89%) showed a beaded (examples shown in Figures of 2C and 4C), three (16.67%) showed a serpiginous tubular (examples shown in Figures of 1C, 1D, and 3B), two (11.11%) cases showed a nodular, two (11.11%) showed a tortuous linear, and one (5.6%) showed a comma shaped enhancements. Among different enhancement shapes, the all ring lesions were the smallest with a mean diameter of less than 2.0 cm while the all-tubular lesions had the longest mean diameter of 6.1 cm (an example shown in Figure 3B).

### MR follow-up

The follow-up MR scans between one to seven months after discharge of the patients were performed in eight patients. For three cases, the locations and shapes of the enhancement lesions had been changed (an example shown in Figures of 2C and 2E). For two cases, the lesions had increased in ring numbers without changing the ring size. For two cases, the images from the follow up scans of three months demonstrated cerebral atrophy with decrease in sizes of the ipsilateral cerebral gyrii and increase in sizes of cerebral sulcii, fissures and ventricles (an example shown in Figures of 4A, 4B and 4C). The MRA image also showed thinning of corresponding cerebral artery and reducing of branches of vasculature (Figure 4D). There was no obvious change for the lesion area in the images of one case.

### Comparisons on the results of surgery, pathology and MR presentations

Among the four cases with a craniotomy, we found a white linear worm in each of two cases. One was a 5.0 cm long and 1.5 cm wide, and another was a 13 cm long and 2.1 mm wide. One of these two worms was still alive after the surgery. The histopathological results showed the worms having a solid-body with characteristic body walls but without a bladder. The body of the larva contained rounded or oval-shaped calcareous corpuscles and bundles of longitudinal muscle fibers. The anterior end contained a characteristic groove. In addition, there were several old and new small cerebral abscesses in the region around the larva. The baseline MR images (i.e., the images in the first scan) of this case with the living worm revealed a serpiginous tubular shape for the lesion, and it was slight hyperintensity on a T2WI. The lesion area was in the white matter of the left parietal lobe, and the irregular large patchy area of the edema was in the perilesional region. Enhanced T1WI showed a serpiginous tubular enhancement of the lesion with the longest diameter of 6.1 cm (Figure 3B). After seven months, the MR images in the follow-up scans showed a change in the location and shape of the enhancement of the lesion (Figures of 3B and 3C). The lesion had slightly moved posterior with a comma-shaped and a ring enhancements, however, the size of the enhancements of the lesion did not change much.

After the surgery, the movement of the worm could still be observed. The length of the worm was approximate 13 cm (Figure 3D). A hard lesion with an ill-defined border and a volume of 3 cm × 2 cm × 3 cm was seen during the surgery. On slicing the lesion, a small cystic lesion of about 1.0 cm × 1.0 cm × 0.7 cm was seen. The cavity with a wall thickness of about 2.0 mm contained a linear yellowish body and partly necrotized substance. The pathologic results showed that cavitations resulted from fibroplasias of cerebral tissue with degenerated larva, and necrotized and calcified substances filled in the cavities. There was fibrosis of surrounding brain tissue with several granulomas infiltration of inflammatory cells and gliosis (Figure 5D). The lesion on T2W images appeared as inhomogeneous. The lesion was slight hyperintensity with edematous areas of irregular perilesional large patchy on enhanced T1W images. In Figure 5C, the lesion areas were shown as multiple ring enhancements.

## Discussions

### Epidemiology on cerebral sparganosis in children

Usually human being is not infested by an adult spirometra mansoni. However when the adult spirometra mansoni infests human gastrointestinal tract, the mechanical and chemical stimulation from the worm can cause discomfort in middle and upper abdomen, dull pain, nausea and vomiting. Sparganum, the larval form of spirometra mansoni, can lead to the sparganosis in human being, and the harm well exceeds that caused by the adult worm. Degree of severity depends on its migration and infected area. Infected areas of the most commonly seen are eyes, subcutaneous tissue of the limbs, oral cavity, face and internal organs. Eosinophilic granulomas can be formed in these areas causing local swell and abscess. If a sparganum infects a brain, then the disease is known as cerebral sparganosis. For human being, there are two pathways of the infection: either by entering through skin of spargana and procercoids or by entering through the mucosa from ingestion of spargana and procercoids.

According to our data and literature [6] there are three most common sources for the infection of the ingestion. The first is for patients to utilize frog meat as poultices to heal wounds or abscesses in the eyes, cheeks and genitals. If the frog meat contains spargana, they can enter through the wound or normal skin or mucosa into the patients’ bodies. Some patients ingested either living tadpoles to heal sores and pain or uncooked and or improperly cooked frog, crabs, snake, chicken and or pig meat. The ingested sparganum passed through the intestinal wall, entered the peritoneum and moved to other places including a brain. The third is for patients infected by untreated water. This provided an opportunity for cyclops to enter the patient bodies. Among our cases, six had experienced high risk factors, such as a history of eating uncooked frog meat or ingesting uncooked crabs or drinking contaminated water.

The literature [6] and our study explored that incidence of a cerebral sparganosis in children and young age adults was higher than the one in middle and old age adult. The possible reasons are the followings: the children and young people usually had more chances to get skin injury and ate infected cyclops water; because the immune system of children and young people was not intact, the sparganum and procercoid invading into their bodies could be survived easier; the blood brain barrier of children and young people was immature, so the sparganum and procercoid invading their bodies were more accessible to their brains.

### Clinical presentation

The clinical presentation of the patients was similar to the one of the patients with brain tumors. In our cases, nine patients presented with headache, eight with convulsions, five weakness in the limbs, two with dizziness, one with projectile vomiting, two with blurred vision, one with intermittent mouth twitching, two with the limb numbness, and one with paralysis. One also had liver sparganosis and presented with headache and right hypochondriac pain.

### MR imaging presentations

Recent publications illustrated importance of imaging modalities in diagnosis of this rare disease [11,12]. Both Song [2] and Moon [6] reported cerebral sparganosis with the following imaging characteristics: low density on CT images; slight hypointense in T1W images and hyperintense in T2W images; a ring or a beaded enhancement on enhanced MR images with a similar shape as sparganum; and without the signal from small punctuate calcification while with the signal from small signal voids in T1W images. Chen [13] reported low and high density lesions on CT images and high density for calcification on CT images and a ring or a beaded or a tortuous enhancement representing the shapes of the parasite on enhanced CT or MR images.

In our cases, MR presentations of cerebral sparganosis in children included solitary or multiple asymmetrical lesions, but a single lesion is a main presentation. Lesions mainly occur in the frontal or parietal lobe, with fewer lesions in temporal, occipital, basal ganglia, cerebellum and brainstem. Lesions mostly affect white matter, with fewer lesions in gray-white matter junction and gray matter. The shapes of lesions include irregular patchy, serpiginous tubular and tortuous linear pathways. Irregular large patchy areas of edema can be appreciated in the cerebral parenchyma surrounding the lesions. They are slight hypointense on T1WI but moderately hyperintense on T2WI with irregular large patchy areas of edema signal. In enhancement images, most lesions show a ring, a beaded, and a serpiginous tubular enhancements. Nodular, tortuous linear and comma-shaped enhancements can also be appreciated in few cases. The MR presentations in our study are similar to those in previous studies [6,11]. However serpiginous tubular and comma-shaped enhancements of lesions have not been previously reported. The major possible reasons for existing different shapes on the lesion images are the followings: a ring, a serpiginous tubular, a tortuous linear, and a comma shaped enhancements are caused by different configuration of worm bodies; tortuous linear and comma shaped enhancements are due to compacting twist of the worm bodies; serpiginous tubular enhancement is due to losing twist of a worm body; and beaded enhancement is formed by eosinophilic granuloma yielded by worm stimulating brain tissue and tunnel produced by the worm body. The followings are a summary of the MR presentations on cerebral sparganosis in children from this study. First, there were a serpiginous tubular, a beaded and a tortuous linear enhancements of the lesions in the images. Second, a ring-enhanced lesion was relatively small with a diameter less than 2 cm, which may be due to sparganum stimulating brain tissue leading to eosinophilic granuloma shown in images. Third, there were changes in the location and shape of the lesions on follow-up enhanced images, suggesting that a migration happened and that the sparganum was alive [14].

A sparganum cannot reproduce in brain tissue and develop into an adult. However, it can survive for a long time and has a good mobility. This causes local inflammation and forming single or multiple eosinophilic granulomas. Often, there is a cavity formed in the granuloma, this may contain one or two sparganums. During dying, the sparganum releases toxins stimulating brain tissues, and generates edemas. The last, in follow-up images, no obvious increase in size was noted in these lesions, but there was an increase in number of enhancement areas. This may be due to movement of a sparganum to other places producing new granulomas.

If the above characteristics exist in the MR images, then the disease: cerebral sparganosis should be considered. We need to use the method of ELISA [10] to further test cerebrospinal fluid and blood serum for sparganum antibodies (i.e., with positive results) for a final diagnosis. Hence, the above-summarized information obtained from the baseline MR scans and follow-up scans are important for diagnosing cerebral sparganosis in children.

Finally, we need to address two other diseases with similar presentations as the ones of cerebral sparganosis in children. The first is parasitic granulomatous cerebrates, such as paragonimiasis, toxoplasmic encephalitis. The MR images of parasitic granulomatous cerebrates also show: hypointense in T1WI but hyperintense in T2WI with perilesional irregular large patchy areas of edema; a ring and or a nodular enhancements; either single or multiple lesion(s) with mass effect; in addition, migratory of the lesions in some cases. Hence, cerebral sparganosis in children can be easily confused with paragonimiasis, toxoplasmic encephalitis and other parasitic granulomatous cerebrates if diagnosis is only made through MRI. However, cerebral sparganosis of MR images also demonstrates specific charateristics: a serpiginous tubular, or a beaded, or a tortuous linear enhancement. A final diagnosis for cerebral sparganosis should be made in combination with using parasite specific immunological assays, such as ELISA, to test related antibodies [7]. The second is gliomas or metastatic tumors. In our series, four of these 18 patients were misdiagnosed as a gliomas or a metastatic tumor. The differences in diagnosis of cerebral sparganosis and a brain tumor should be the followings: a cerebral sparganosis can appear as a serpiginous tubular, or a beaded, or a tortuous linear enhancements while the images of brain tumors do not possess such enhancement patterns; changes in location and shape of the enhancements in follow-up MRI are only noted for cerebral sparganosis; a size of ring enhancement for cerebral sparganosis is relatively small (less than 2 cm diameters), and it is almost a constant in the follow-up images while the size of a brain tumor usually is relatively large, and becomes bigger in the follow-up images; and magnetic resonance spectroscopy (MRS) can also be applied to differentiate a cerebral sparganosis from a brain tumor patient [15,16]. The main treatment of cerebral sparganosis in children is a surgical resection. For the surgery, care should be taken to totally remove the worm for avoiding recurrence. Recently the authors in [17] concluded that priority should be given to image-guided stereotactic aspiration since it causes the smallest wounds. Praziquantel and albendazole used for deworming are not very effective for the disease. Prevention should rely on education. Children and parents should not use frog meat as poultices and avoid ingestion from eating uncooked meat and drinking untreated water.

## Conclusions

The MR presentations of cerebral sparganosis in children were presented as slight hypointensity on T1-weighted images and moderate hyperintensity on T2-weighted images with a perilesional brain parenchymal edema. Enhanced MRI features are rich including a ring, and or a beaded and or a serpiginous tubular shape. The MR presentations in our study are similar to those in previous studies. However, serpiginous tubular and comma-shaped enhancements of the lesions have not been previously reported. Enhanced and MRI follow-up scans are the most valuable for providing the foundation of the clinical diagnosis of cerebral sparganosis in children.

## Abbreviations

CT: Computer tomography; ELISA: Enzyme-linked immunosorbent assay; MRA: Magnetic resonance angiography; MRI: Magnetic resonance imaging; T1WI: T1 weighted image; T2WI: T2 weighted image.

## Competing interests

The authors declare that they have no competing interests.

## Authors’ contributions

All authors approved the final manuscript CG carried out acquisition of data, analysis and interpretation of data, and drafted the manuscript in Chinese, and helped to write the first English manuscript. WL participated in the design of the study, performed analysis of MR images, and revised and translated the Chinese manuscript to the English. AC helped to draft the first English manuscript. *XW is one of the corresponding authors. He conceived of the study, and participated in its design and led the cooperation. *BH is another corresponding author. He did literature review, performed data analyses, answered the questions from reviewers, wrote the final manuscript and led the cooperation.

## Pre-publication history

The pre-publication history for this paper can be accessed here:

http://www.biomedcentral.com/1471-2431/12/155/prepub
